# Gender dysphoria and psychosis - symptom or comorbidity?

**DOI:** 10.1192/j.eurpsy.2025.2298

**Published:** 2025-08-26

**Authors:** B. V. Ferreira, C. Alves, J. F. Fontes, M. M. Serra, T. Pereira, R. R. Henriques, M. Gonçalves

**Affiliations:** 1 Clínica 5; 2 Clínica 3; 3 Clínica 2, ULS São José - Hospital Júlio de Matos, Lisboa, Portugal

## Abstract

**Introduction:**

Gender dysphoria (GD) refers to the marked incongruity between the experience of one’s gender and the sex at birth.

Psychiatric illness is one of the major negative prognostic features for the outcome of GD. In particular, much controversy exists regarding whether individuals with psychotic symptoms should receive gender affirmative treatment.

**Objectives:**

The present review aims to provide an overview of the current literature of the occurrence of gender dysphoria symptoms during psychotic episodes and identify challenges in differential diagnosis and treatment and offering recommendations to overcome them.

**Methods:**

Articles published on the last five years were searched on Medline, PubMed, Web of Science and Springer Link using the following keywords: gender identity, psychosis, gender dysphoria.

**Results:**

Delusions about one’s physical appearance and the desire to change the body can be observed in patients with schizophrenia or other psychotic disorders. Therefore, the differential diagnosis is critical for therapeutic planning and effort is required to carry out the necessary diagnostics when articulation of gender dysphoric feelings coincides with the onset of psychosis. It is crucial to evaluate the chronology and dynamics of the individual symptoms, their constancy, patient’s criticism, and response to antipsychotic treatment.

**Conclusions:**

Some presentations of GD can be created by life experience in individuals who have underlying mental or neurophysiological abnormalities. The experiences of practitioners indicate that coincidence of schizophrenia and gender identity disorder is possible, that is why the differential diagnosis needs time, careful observation, examination and cooperation of psychiatrist and sexologist.

This review addresses the significance of understanding each patient’s individual circumstances, focusing specifically on proper physician training and direct patient care.

**Disclosure of Interest:**

None Declared

